# Policy makers, genetic engineers, and an engaged public can work together to create climate-resilient plants

**DOI:** 10.1371/journal.pbio.3002208

**Published:** 2023-07-13

**Authors:** Bella N. Archibald, Vivian Zhong, Jennifer A. N. Brophy

**Affiliations:** Department of Bioengineering, Stanford University, Stanford, California, United States of America; University of California, Davis, UNITED STATES

## Abstract

As climate change affects weather patterns and soil health, agricultural productivity could decrease substantially. This Perspective argues that synthetic biology could be used to enhance climate resilience in plants and create the next generation of crops, if the public will accept it.

## Teaser

As climate change affects weather patterns and soil health, agricultural productivity could decrease substantially. Synthetic biology can be used to enhance climate resilience in plants and create the next generation of crops, if the public will accept it.

**Fig 1:**
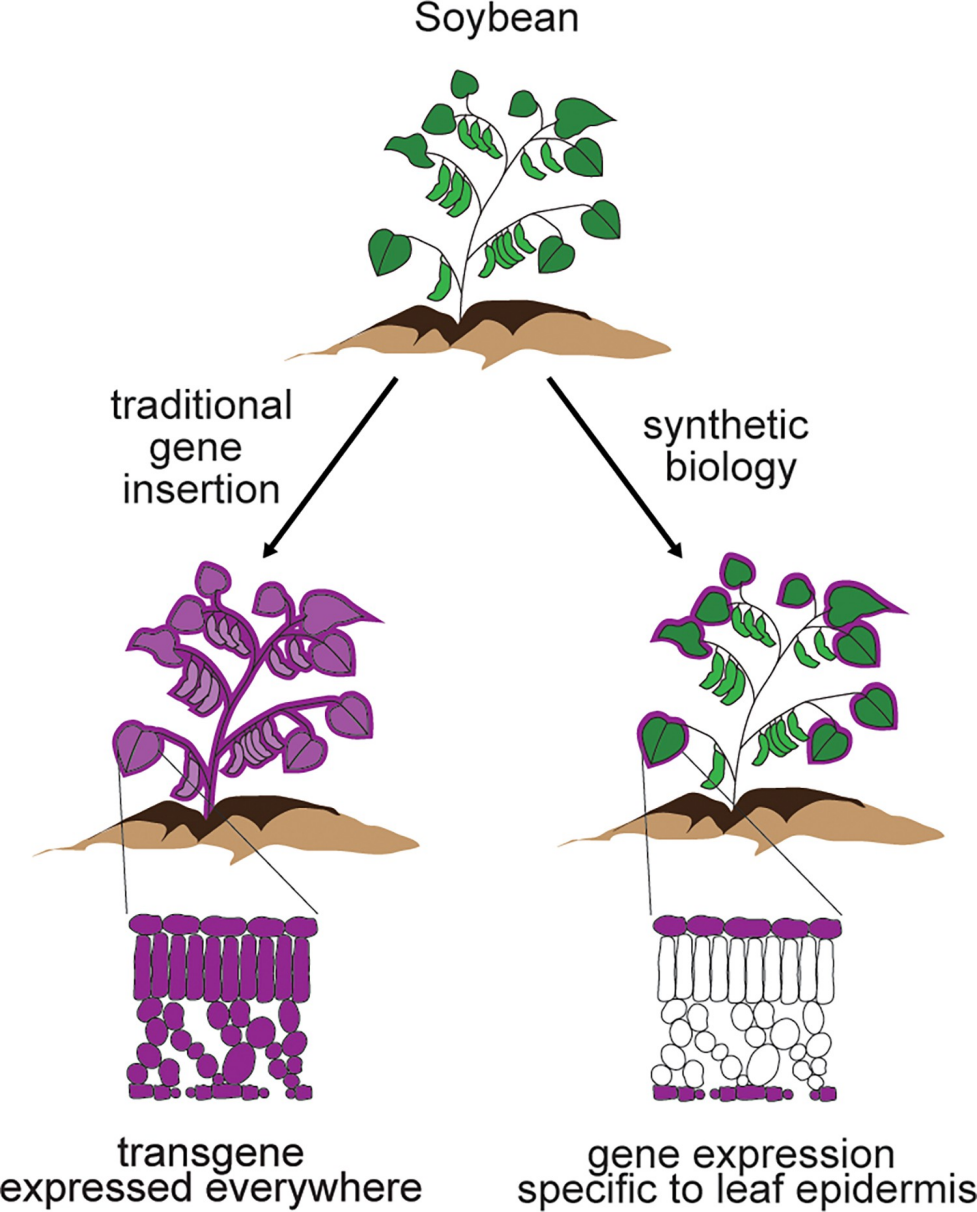
Using synthetic biology to engineer specific plant traits. Traditional genetic engineering of soybean (left) is compared to a synthetic biology approach (right). In this example, the plants are engineered to produce anti-pathogen compounds such as BT (purple) in the whole plant (traditional engineering) or in leaf epidermal cells (synthetic biology) where the compound can prevent disease without changing food quality.

Climate-resilient plants will be essential for agricultural stability in a less predictable world. As climate disruptions such as droughts, floods, and extreme temperature fluctuations become more common, current farmland will become less productive. Each one-degree Celsius temperature increase is predicted to decrease wheat, rice, and maize yields by 6%, 3%, and 7%, respectively [1]. Thus, in the most drastic climate scenarios, grain production could fall by 15% to 35% over the next decade. Resilient crops will be an important part of ensuring future agricultural stability. However, plants that can tolerate extreme environmental stress, including varieties with better water-use efficiency, heat and flood tolerance, and frost resistance, will not be easy to generate using established methods. Breeding and random mutagenesis are too slow and can be difficult to control. Even CRISPR-Cas9-based gene editing is likely to be insufficient because improved resilience will probably require dynamic and/or tissue-constrained modifications to plants (Fig 1).

Synthetic biology—a field of advanced genetic engineering that aims to introduce new capabilities into living organisms—has the potential to rapidly develop climate-resilient crops. In contrast to standard crop engineering, in which single genes from other organisms (e.g., viruses, bacteria, or mammals) are introduced into plants and expressed in all cell types, synthetic biology can be used to express many genes in a more controlled manner; for example, only in specific leaf or root cells or in response to environmental changes (Fig 1). This precise genetic control allows synthetic biologists to engineer new, complex behaviors into living organisms. When applied to plants, synthetic biology could be used to change the way crops respond to the environment while maintaining their desirable features, such as fruit size, nutritional content, or stem height. For example, synthetic biology could be used to change root growth in dry soils in order to enhance drought tolerance. This improved environmental responsiveness may help plants adapt to extreme weather and reverse the detrimental byproducts of domestication, which often improves yield traits at the expense of environmental resilience [2].

Unfortunately, negative consumer sentiment may limit the ability of genetically modified (GM) plants to enhance agricultural sustainability. Here, we define GM plants as having genetic material altered by humans through means other than breeding (mating); this includes everything from chemical mutagenesis and CRISPR-Cas9-induced deletions to insertion of multiple foreign genes. Though the definition and perception of GM plants are constantly in flux, current research shows widespread GM hesitancy. A survey in 2020 found that approximately half of US adults are wary of the health effects of GM foods [3]. In Europe, anti-GM sentiment is even stronger [4]. These attitudes have global ramifications; India recently banned pest-resistant eggplants, which have boosted yields and decreased pesticide use in Bangladesh and the Philippines, because of anti-GM sentiment. Similarly, several African countries have been reluctant to approve GM crops that increase yield despite a clear link between agricultural productivity and malnourishment [5]. These examples show that innovative and promising technical advances will not matter if consumers refuse to accept GM plants.

Confusion around GM terminology and regulatory status may contribute to the negative public perception of GM foods. Recent social science research found that using concrete language and examples when discussing GM products increased positive emotions and support for GM applications and GM in general [6]. Thus, regulatory frameworks that separate crops by improvement method (e.g., CRISPR editing versus chemical mutagenesis) may contribute to distrust because they can be difficult to understand. We are in favor of regulations that focus on the end product and potential impact on the world—environmentally, economically, socially, and for human health—rather than the method used to modify the plants’ genetics. New research into the public perception of GM plants, stratified by trait and method, would help to update our understanding of consumer sentiments and determine whether or not trait-based regulation would be palatable.

While there are currently no crops on the market that contain the more complex genetic modifications that are possible with synthetic biology, regulatory frameworks could play a key role in directing technology development. In 2020, the United States of America stopped regulating the import, movement, and environmental release of GM plants containing minimal genetic changes and no foreign DNA [7]. They also revised regulations for plants containing DNA from other organisms. These changes have accelerated the development of gene-edited crops [8]. In a significant breakthrough for GM crops, the Philippines recently became the first country to approve the cultivation of “Golden Rice,” which has been genetically modified for enriched vitamin content through the introduction of 2 foreign genes (a plant phytoene synthase and a bacterial carotene desaturase) [9]. This year, the United Kingdom and China moved to deregulate crops created with gene-editing tools, such as CRISPR, which could pave the way for greater GM permittivity [10,11]. Unfortunately, crops produced through the introduction of foreign DNA remain regulated in the UK and China, and there are prominent cases in which GM regulation is becoming stricter; for example, Mexico published a presidential decree banning GM corn in 2020 [12]. Shifting, heterogeneous regulations may be one reason that growth of the field of plant synthetic biology has been slow. We hope that the development of plants with the potential to significantly enhance agricultural sustainability begins to shift regulatory frameworks in favor of GM crops and initiates a positive feedback loop of technology and crop improvement.

Because sustainability and climate resilience are complex goals, we believe scientists should carefully evaluate all potential solutions to identify the most effective path forward. For example, flooding is predicted to become more prevalent as the climate changes. On flooded farms, plants can quickly become water-logged and oxygen-starved, leading to mass crop loss. One way to increase flood tolerance in plants could be to make plants that deposit more suberin (a waxy compound that can protect plant roots) in response to flooding. However, installing water pumps to drain crop fields could also accomplish the same goal; with multiple solutions to a problem, one must compare all costs and benefits. Biology-based solutions may be cheaper and faster in many instances, but plant scientists, economists, policy makers, and engineers need to work together to critically consider potential approaches to ensure that we undertake the fastest, easiest, fairest, and most impactful responses to climate change.

Ultimately, the public and policy makers must decide how to deploy plant synthetic biology to fight climate change. By putting financial incentives in place, policy makers could promote the development of sustainability-oriented crops. Increased governmental or philanthropic funding for plant synthetic biology could lower the barrier to entry for academic researchers and small agricultural biotech companies, thereby substantially increasing the diversity of players in the field. Ideas for incentivizing agricultural innovation have been brought forward before. In 2008, China launched the Major Breeding Projects of GMOs Varieties (GMMP), the largest public investment specifically for GM biotechnology research and development, with an aggregate public budget that reached 3.8 billion USD by 2020. In a similar vein, the US Congress authorized AgARDA in 2018. AgARDA is a US Department of Agriculture-housed Agriculture Advanced Research and Development Authority designed to enable “moonshot” projects in agriculture. These high-risk, high-reward projects could significantly push forward the development of engineered crops to revolutionize agricultural sustainability. However, AgARDA remains unfunded. It is time to support more crop innovation and to enable synthetic biology to help usher in agricultural change. We believe plant synthetic biology has an important role in the development of a sustainable world.
